# An LSTM and GRU based trading strategy adapted to the Moroccan market

**DOI:** 10.1186/s40537-021-00512-z

**Published:** 2021-09-24

**Authors:** Yassine Touzani, Khadija Douzi

**Affiliations:** Computer Lab of Mohammedia, Faculty of Science and Technology of Mohammedia, Hassan II university, Mohammedia, Morocco

**Keywords:** Deep Learning, Stock market, Trading strategies, Financial times series

## Abstract

Forecasting stock prices is an extremely challenging job considering the high volatility and the number of variables that influence it (political, economical, social, etc.). Predicting the closing price provides useful information and helps the investor make the right decision. The use of deep learning and more precisely of recurrent neural networks (RNNs) in stock market forecasting is an increasingly common practice in the literature. Long Short Term Memory (LSTM) and Gated Recurrent Unit (GRU) architectures are among the most widely used types of RNNs, given their suitability for sequential data. In this paper, we propose a trading strategy designed for the Moroccan stock market, based on two deep learning models: LSTM and GRU to predict the closing price in the short and medium term respectively. Decision rules for buying and selling stocks are implemented based on the forecasting given by the two models, then over four 3-year periods, we simulate transactions using these decision rules with different settings for each stock. The returns obtained will be used to estimate an expected return. We only hold stocks that outperform a benchmark index (expected return > threshold). The random search is then used to choose one of the available parameters and the performance of the portfolio built from the selected stocks will be tested over a further period. The repetition of this process with a variation of portfolio size makes it possible to select the best possible combination of stock each with the optimized parameter for the decision rules. The proposed strategy produces very promising results and outperforms the performance of indices used as benchmarks in the local market. Indeed, the annualized return of our strategy proposed during the test period is 27.13%, while it is 0.43% for Moroccan all share Indice (MASI), 15.24% for the distributor sector indices, and 19.94% for the pharmaceutical industry indices. Noted that brokerage fees are estimated and subtracted for each transaction. which makes the performance found even more realistic.

## Introduction

The semi-strong form of market efficiency hypothesis [1] which states that security prices react very quickly to publicly available new information is commonly accepted in the financial field. Furthermore, market data are extremely volatile and noisy, as a result, predicting stock prices is a notoriously challenging task. However, there is a slew of market anomalies that contradicts the efficient market theory [2], hence the need for a trading strategy to better trade stocks able to outperform the market. Accurate forecasts provide valuable information to investors and allow them to adjust their position (buy, hold or sell) according to the price trend, it also allows building a winning trading strategy. This will explain the large number of works published in this field in recent years. In all of these articles, we find a lack of works devoted to the Moroccan market. In this paper, We propose a new trading strategy tailored to the Moroccan market comprising two parts: the first part for forecasting and the second is dedicated to decision rules for buying and selling stocks. The proposed strategy will be the first strategy designed for the Moroccan market. Unlike the American or European market where almost every day we have an interesting trade with a high return expectation. The Moroccan market is characterized by its occasional opportunities. The proposed strategy address this problem and guarantees a high return even in a crisis period (impact of COVID 19 on the Moroccan stock market). The results of the proposed approach outperform the set of indices used in the local market as a benchmark for evaluating the performance of financial products such as Undertaking for Collective Investments in Transferable Securities (UCITS). The rest of this article is structured as follows: In the related work section, we go through a non-exhaustive list of previous work using LSTM and GRU in several areas, and then we focus on articles using both architectures for stock price forecasting. In the following section, we provide a more detailed overview of the proposed trading strategy components. The experimental results section will be dedicated to the experiments as well as the results obtained, accompanied by the discussion section in which we comment on the results obtained. Finally, this paper ends with a conclusion and future work.

## Related work

Machine learning techniques [3] are used in a variety of fields. Deep learning is one of its most popular techniques, particularly for time series problems using recurrent units (mainly LSTM and GRU) perfectly suited to the sequential nature of the data. In fact, LSTM and GRU architectures showed high performance for forecasting tasks in several fields like healthcare, transportation, finance as well as others. Shahid et al. [4] evaluated different machine learning and deep learning models to predict confirmed cases, recovered cases and death cases for the COVID 19 pandemic. They found that LSTM, GRU and Bidirectional LSTM (Bi-LSTM) provided accurate predictions for all three time series. ArunKumar et al. [5] propose RNN-LSTM and RNN-GRU models for a 60-day forecast of the covid19 pandemic. For the prediction of confirmed cases, the authors conclude that LSTM model perform quite well for countries like the United States, Brazil, South Africa, and Iran. The GRU model has better performance for countries like India, Russia, Mexico, and the United Kingdom. Wang et al. [6] Proposed a new approach for truck traffic flow prediction, paper authors noticed that LSTM and GRU have better performance compared to existing approach based on support vector machine (SVM) or Autoregressive integrated moving average (ARIMA). Regarding financial time series modeling, the number of papers, including LSTM and GRU is very important. Liveris et al. [7] present a CNN-LSTM model in forecasting gold price time series. The proposed model was compared to advanced deep learning and classical machine learning approaches and it turns out that it provides the best performance: the mean absolute error (MAE) and the root mean squared error RMSE are respectively 0.008 and 0.09 (gold price variable is ranging from 100$ and 134$). Liu et al. [8] Proposes a new hybrid system for one day ahead forecasting, closing price data will be decomposed into several components using Empirical Wavelet Transform (EWT) algorithm [9], LSTM predictors with a dropout are built to predict the decomposed closing price time series, LSTM model hyperparameters are selected using the particle swarm optimization (PSO) algorithm [10]. Error correction is carried out using the outlier robust extreme learning machine (ORELM) method. Finally the LSTM forecasts and the ORELM error forecasts are added to produce the final closing price prediction. The proposed framework produces excellent results (mean absolute percentage error (MAPE) $$\approx 0.15\%$$). In [11] Althelaya et al. used GRU and LSTM units to build a stacked and bi-directional architecture for short term (1 day ahead) and long term (30 day ahead) forecasting of the SP500 index. The results of the GRU and LSTM models are very close and outperform the multilayer perceptron (MLP), which was also used in this study. In [12] Patel et al. propose a model incorporating LSTM and GRU for one, three, and seven days ahead prediction of Litecoin and Monero cryptocurrency. In order to build a threshold based portfolio Lee and Yoo [13] develop three types of RNN models : classical RNN, LSTM and GRU to forecast one month ahead, the top ten stocks in Standard and Poor’s 500 index using monthly data (OHLCV: open,high,low,close and volume). They conclude that the LSTM outperformed the two other architectures. In [14] Cao et al. propose a new hybrid financial time series forecasting, decomposition part is carried out using empircical mode decomposition (EMD) [15] and complete ensemble empirical mode decomposition with adaptive noise (CEEMDAN) [16], an LSTM model is then trained on the intrinsic mode function (IMF) including the residual component, the final forecast is obtained by adding the set of predictions obtained for each component. Despite the wide availability of financial forecasting papers, there is no model adapted to the Moroccan market in the literature; however, many works have been proposed dedicated to other markets. Li et al. [17] propose a framework for predicting the Hong Kong stock market. The proposed framework incorporating stock market data and news sentiment. Technical analysis is used to represent stock prices. Sentiment analysis is used to represent news information, then the sequential representation is fed to an LSTM model for the prediction task. The proposed model outperforms SVM and Multiple Kernel Learning (MKL) used as a benchmark. In [18] Budiharto propose an LSTM-based approach for stock price forecasting in Indonesia. Yadav et al. [19] Propose an optimized LSTM for Indian stock market forecasts. The Sri Lanka market was the subject of an RNN model proposal by Samarawickrama et al. in [20] Nti et al. [21] use artificial neural network (ANN) to predict future movement of stock price in Ghana for 1, 7, 30, 60 and 90 days ahead based on public opinion. Papers cited above demonstrated that both of LSTM and GRU models perform brilliantly in financial time series forecasting. We will also use them for our proposed approach.

## Proposed model

In this paper, we propose a new trading strategy tailored to the Moroccan market, based on two deep learning models. LSTM model provides short term prediction while GRU model predicts the medium term. Once the predictor component provides the price forecasts, suitable decision rules for each stock should be established to complement the proposed strategy. Before going in depth of the proposed approach, we will give a brief overview of LSTM and GRU architectures.

### Long short term memory

Recurrent Neural Networks RNNs are a type of neural network widely used in the field of deep learning [22–24], it turns out that classical RNNs are extremely difficult to train to handle long term dependency [25] because of gradient vanishing problem (gradient exploding can occur also, but very rarely). To overcome the vanishing gradient problem LSTM is proposed initially by Hochreiter et al. [26], then improved by Gers and Schmidhuber [27]. The LSTM unit is the most basic component of an LSTM architecture; it’s a series of gates and cells that work together to produce a final result. The forward pass of an LSTM unit is modeled by Eqs. (1–6)

1 $$\begin{aligned} f_t= & {} \sigma (W_f.x_t+U_f.h_{t-1}+b_f), \end{aligned}$$

2 $$\begin{aligned} i_t= & {} \sigma (W_ix_t+U_ih_{t-1}+b_i), \end{aligned}$$

3 $$\begin{aligned} \widetilde{c_t}= & {} tanh(W_cx_t+U_ch_{t-1}+b_c), \end{aligned}$$

4 $$\begin{aligned} c_t= & {} f_t * c_{t-1} + i_t * \widetilde{c_t}, \end{aligned}$$

5 $$\begin{aligned} o_t= & {} \sigma (W_ox_t+U_oh_{t-1}+b_o), \end{aligned}$$

6 $$\begin{aligned} h_t= & {} o_t * \tanh (c_t), \end{aligned}$$

where $\sigma$ is sigmoid function, $f_t$ is forget gate activation vector, $i_t$ input gate activation vector, $o_t$ output gate activation vector, $\widetilde{c_t}$ cell input activation vector, $c_t$ cell state, $h_t$ output vector of LSTM unit, all W and U are weights, b is biases vector and symbol * for Hadamard product (element wise product). Weights W, U and biases b are to be learned during the training process. In order to better decipher the equations cited above, let’s start with cell state $c_t$, it contains two kinds of information: old information to keep from past state $c_ {t-1}$ specified using forget gate, it decides the percentage of information to keep by computing a value between 0 (throw completely) and 1 (keep completely), and new information to include in the cell state calculated using input gate $i_t$ and cell activation $\widetilde{c_t}$ which are computed using (2) and (3), respectively. Finally the calculation of the final output value is performed in two steps. A potential value is calculated using (5) for the first time. This value will be regulated using the information present in cell state as indicated in (6). The use of the cell state in the final calculation makes the LSTM powerful in tasks where information must be stored and used later (long term). Language modeling is a simple example of this situation. The verb conjugation in the middle or even the end of the sentence depends on the subject at the beginning of the sentence.

### Gated recurrent unit

Gated Recurrent Unit GRU was introduced in 2014 by Cho et al. [28] To solve the vanishing gradient problem experienced by classical recurrent networks. Same as LSTM, the input value interacts with the information from the previous state to calculate the different values of intermediate gates which will subsequently be used to decide on the value to be output. The forward pass of a GRU unit is modeled by Eqs. (7–10)

7 $$\begin{aligned} z_t= & {} \sigma (W_z.x_t+U_z.h_{t-1}+b_z), \end{aligned}$$

8 $$\begin{aligned} r_t= & {} \sigma (W_rx_t+U_rh_{t-1}+b_r), \end{aligned}$$

9 $$\begin{aligned} \widetilde{h_t}= & {} tanh(W_hx_t+U_h(r_t*h_{t-1})+b_h), \end{aligned}$$

10 $$\begin{aligned} h_t= & {} (1-z_t)*h_{t-1}+z_t*\widetilde{h_t}, \end{aligned}$$

where $\sigma$ is sigmoid function, $z_t$ is update gate activation vector, $r_t$ reset gate activation vector, $\widetilde{h_t}$ candidate vector and $h_t$ is the output vector of GRU unit. W and U are weights, b is bias vector and symbol * for Hadamard product. Same as for LSTM, weights and biases are to be learned during the training process. Let us go deep through the equations cited above to better understand how a GRU unit works. First update gate is computed using input vector $x_t$, output of previous unit $h_{t-1}$ and sigmoid activation function. Reset gate is calculated in the same way as update gate using its own weights and bias. The reset gate is then involved in the candidate value calculation. It determines how much information from the previous state should be preserved. Indeed, from Eq. (9) we can notice that with an $r_t$ value very close or equal to zero, only the input value will be considered in candidate value calculation. Finally, the output value is calculated by calibrating the previous output and the new candidate output. The calibration is carried out by the updating gate $z_t$. ($z_t=0$ copy the previous output, $z_t=1$ generate a new output regardless of the old output). We can observe a kind of similarity between LSTM and the GRU, indeed both implement an intermediate gating mechanism used later in the output value calculation. Regarding the two models performance, Chung et al. [29] demonstrated that GRU outperforms LSTM in a number of tasks, this observation is also shared by Jozefowicz et al. [30] who found that GRU outperforms LSTM in a number of tasks except language modeling. On the other hand, Shewalkar et al. [31] finds that the LSTM outperforms GRU in the speech recognition task. However, they confirm that the GRU optimization is faster. In general, both LSTM and GRU are powerful and well-suited to sequential data. The GRU has the benefit of faster optimization compared with an LSTM because it has less parameters.

### Prediction component

**Fig. 1:**
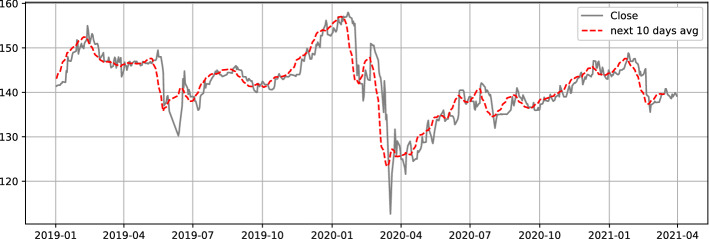
Price with next days Moving average for IAM stock

**Fig. 2:**
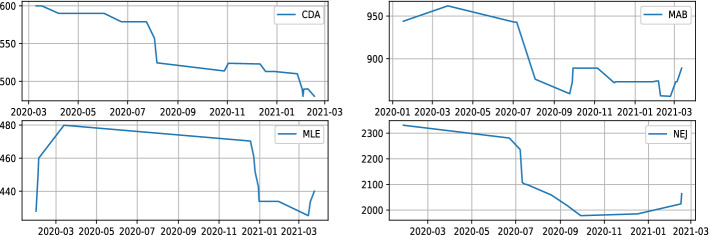
Example of stocks with trading discontinuity

**Fig. 3:**
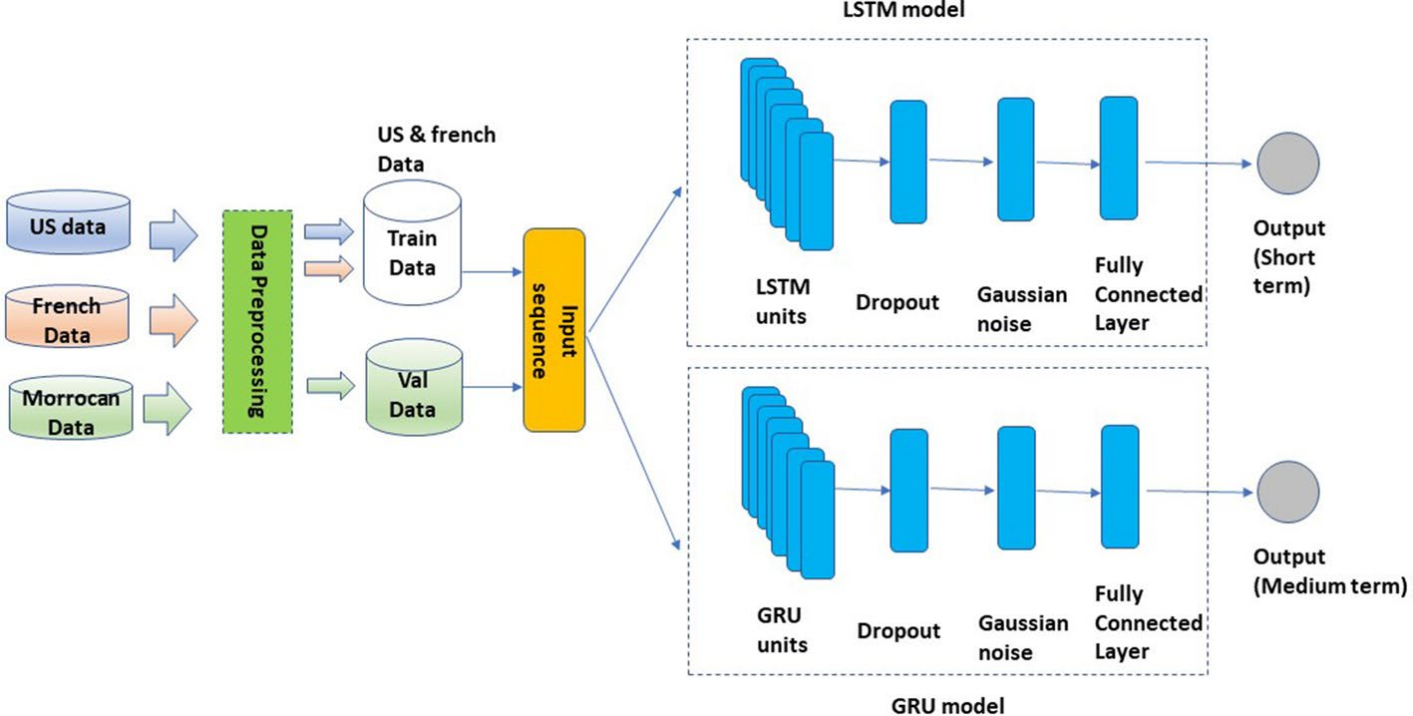
Proposed LSTM and GRU models architecture

This part aims to forecast short and medium term price pattern, a high forecasting accuracy will lead to a successful trading strategy. We will be interested in the moving average described using the formula (11) to better capture the trend:

11 $$\begin{aligned} y^h_{t}=\frac{1}{h}\sum _{i=1}^{h} y_{t+i}, \end{aligned}$$

where $y_t$ is closing price at time t. We will use the notation $y^h_{t}$ to design moving average of next h days at time t. It will be our target variable we are attempting to forecast. It reduces daily price noise and summarizes well the trend information. Fig. 1 is a more accurate depiction of how the price average captures trend data. Once we identify our target variable we need to deal with data issues. Indeed, the Moroccan market presents peculiarities that distinguish it from other well-developed markets such as the European or American market. The major problem encountered lies in lower liquidity. We observe a discontinuity of exchanges for a large number of shares as shown in Fig. 2. It has often occurred that a stock is neither sold, nor bought for several days or even several weeks or months. Which automatically results in missing data for those trading days. In addition, the Moroccan market only offers 76 stocks for trading [32]. This number associated with the missing data problem reduces considerably the amount of data obtained over a given number of years. It is well known, acquiring a large dataset contributes to enhance the model performance and throw it away from overfitting phenomena [33]. To address the problem of data volume we will use external data from other markets (American and French market). These collected data, make it possible to obtain a large dataset. With regard to the problem of trading discontinuity, we will rule out the stocks that suffer from liquidity issues. The relatively demanded shares on the market will be retained. At this level, we have all the required elements to train our two deep learning models. Data from the US and French market will be used as training data and Moroccan data will act as validation and testing data. Using only historical prices, two recurring networks will be trained to forecast future average closing prices for the short and medium term. The two models are shown in Fig. 3. Although training data was gathered from external markets. The use of local data as validation will allow to adjust the parameters according to the Moroccan market characteristics.

### Parameters tuning

To develop a successful trading strategy, forecasts should be followed by good decision rules. Let ${\hat{y}}^m_{t}$ and ${\hat{y}}^s_{t}$ the predictions given by our predictor component for medium and short term horizon respectively. We define the following ratio:

12 $$\begin{aligned} \theta ={\hat{y}}^m_{t}/y_t, \end{aligned}$$

where $y_t$ is closing price at time t. This ratio indicates how is the medium term prediction compared to the actual price. A value greater than one mean price is expected to rise during the upcoming days. We will try to identify optimal threshold $\theta ^*$, when it is outstripped, the trading is profitable. The methodology used to evaluate the $\theta ^*$ will be detailed below once all the decision rules of the strategy have been described. In the trading field, buying a financial instrument is known as an open position, and selling it is called a close position. We only open a position for our proposed strategy when three conditions are verified:

- $$\theta >= \theta ^*$$.
- ${\hat{y}}^s_{t} > y_t$. where ${\hat{y}}^s_{t}$ is the short term forecasting and $y_t$ is the actual closing price.
- There is no open position with the stock to buy.

The first condition seems quite clear, medium term forecasting should exceed a given threshold (tuned previously for each stock). With the respect to the second condition, it completes the strategy and regularizes open position timing. Indeed, even though if we expect prices to increase in the medium term horizon, price can decrease in the short term horizon before increasing. In this case, we delay the purchase to buy at a lower price. Finally, no open position with the stock to buy for simplification reasons. Regarding closing an open position, two conditions need to be checked:

- $$\theta < 1$$
- $${\hat{y}}^s_{t} < 1.$$

**Fig. 4:**
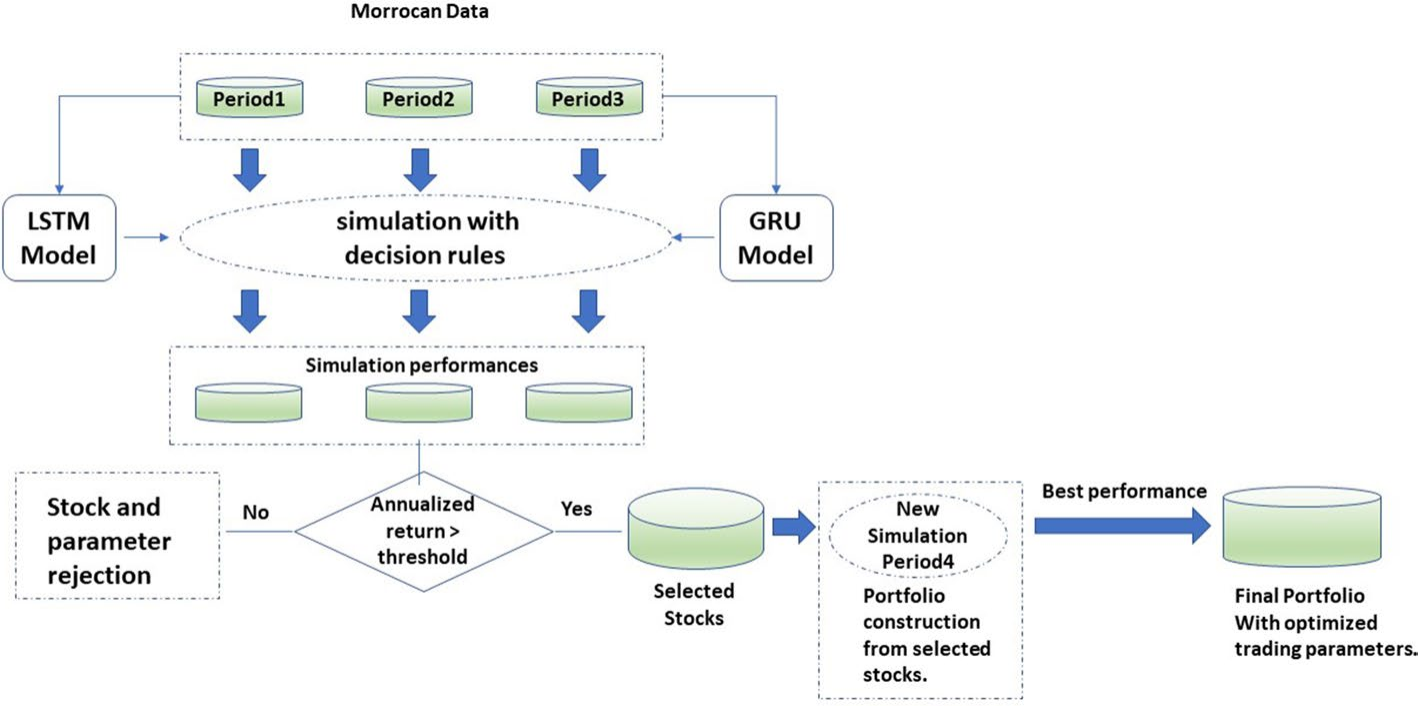
Stock selection process

Concerning close position conditions, it seems more intuitive, we close the position when prediction is less than the current price for short and medium term horizon. To secure trading operation, trader typically sets a stop limit. It is a minimal limit when it’s reached, the operation is immediately closed in order to reduce the losses in case of strong prices drop. Notice that no stop limit is defined in our proposed strategy. It is extremely risky, but the ground truth proves us right, the results obtained support this decision. Now that the strategy is well detailed, we can describe the tuning methodology for $\theta$ parameter. We will simulate the strategy for each stock over periods of equal duration (3 years each, for example). The performance (annualized return) of each stock is tracked using various $\theta$ values. Throughout all simulation times, we record the return achieved by all stocks for each value of the $\theta$ parameter. The returns obtained will be used to estimate the expected return for each configuration (each value of $\theta$ parameter). Then a return threshold is set. We will keep the configurations (stock, $\theta$) that have estimated return greater than the threshold. The selected stocks are then used to build portfolios of varying sizes. To evaluate the performance of the portfolios a simulation over a new period is used. For stocks with multiple $\theta$ parameter values that meet the selection criteria, we will choose $\theta$ at random(several repetitions will be performed to check various $\theta$ values). This whole process will be carried out for a variety of portfolio size. The suggested method is a hybrid between grid and random search. Finally, the shares in the portfolio that has the maximum observed return, will be retained by our strategy. The entire portfolio selection process is depicted in Fig. 4. It is important to highlight that in all the simulations, brokerage fees are estimated and added to buying prices or subtracted from selling prices. Regarding the time complexity of our approach. Let s be the number of stocks, $\Theta$ the number of $\theta$ parameters used during the simulation, and d the number of trading days available during the simulation period. The complexity time is O(s,$\Theta$,d)=s*$\Theta$*d. Given that the number of stocks in the Moroccan market is very limited and the number of parameters $\theta$ can be neglected compared to the number of trading days the complexity of each simulation can be express as O(d) = d. Concerning portfolio construction O (d,I) = d*I where I is the number of stock combinations tested during the random search process.

## Experimental results

### Dataset description

In order to train predictor component models, we will use two sources of data: the first one is data of all stocks in the SP500 index (except BRK.B and BF.B discarded due to data loading error) extracted from yahoo finance using pandas datareader library [34]. The second is data of all stocks in the CAC40 index, collected from investing.com using investpy library [35]. Extracted data are from January 2010 to January 2019. Concerning Moroccan data, to escape the discontinuity issue described in the prediction component section we will be working with stocks that have a relatively high average volume traded in 2019 and 2020. As a result, out of the 76 stocks available, we choose 32. Data from January 2010 to January 2019 will be used as validation data to tune LSTM and GRU predictors parameters. Data from March 2019 to March 2021 will be used to test (hold out dataset) our proposed strategy performances. Note that the Moroccan data are extracted in the same way as the CAC40 data.

### Metrics

We have two parts to evaluate, first we have to evaluate the quality of the predictions provided by both models LSTM and GRU. We will use popular metrics mean absolute percentage error (MAPE), mean squared error (MSE) and root mean squared error (RMSE) given respectively by Eqs. (13–15)

13 $$\begin{aligned} MAPE= \frac{1}{n}\sum _{i=1}^{n} | \frac{y_i-\hat{y_i}}{y_i}|, \end{aligned}$$

14 $$\begin{aligned} MSE= \frac{1}{n}\sum _{i=1}^{n} (y_i-\hat{y_i})^{2}, \end{aligned}$$

15 $$\begin{aligned} RMSE= \sqrt{\frac{1}{n}\sum _{i=1}^{n} (y_i-\hat{y_i})^{2}}, \end{aligned}$$

where y is ground truth variable and ${\hat{y}}$ is its prediction. Regarding the proposed trading strategy, it will also be evaluated using return-oriented metrics. We will use the return given by the following formula:

16 $$\begin{aligned} r=\frac{y_f-y_i}{y_i}, \end{aligned}$$

where $y_f$ and $y_i$ are respectively final sell price and initial buying price. We also use the annualized return to capture the effects of compounding (earnings reinvestment), annualized return is calculated using the below formula:

17 $$\begin{aligned} r_a=(1+r)^{1/years}-1, \end{aligned}$$

where r is return calculated in Eq. (16) and years is the total investment period in years. The wine rate is also used, it is an indicator that gives the winning trade percentage, given by the following formula :

18 $$\begin{aligned} winerate=\frac{winning\_trade}{Total\_trade}. \end{aligned}$$

### Strategy components

We will try 2 types of RNN architectures for the predictor component. For short term prediction, we will train an LSTM model, regarding medium term forecasting, we train a GRU model. All models are designed with Keras API [36], number of layers and unit are determined after a random search. Weights are initialized using He initializer [37] and optimized using adaptive moment estimation (Adam) algorithm [38]. Finally Dropout [39] is used as regularization technique and Gaussian noise is added for better generalization [40]. Once the prediction component is completed, a simulation is run over the following periods from 2010 to 2012, 2011 to 2013, 2012 to 2014 and 2013 to 2015, using a set of values of $\theta$ parameter and for all stocks. We calculate stock return obtained over each period using formula (10). The return is then analyzed using (17) (investment period is 3 years for each period). The top three results for each simulation time, as well as the parameter used, are shown in Table 1. Then a benchmark return is created. We pick the stocks that outperform the benchmark (using estimated return). using random search we try several stocks combination and we choose the combination of stocks that yielded the highest return. Note that the amount invested is the same for each stock. The profit generated from trade is fully reinvested (compounding). Finally the return is calculated at the end of the period using the initial amount and the final amount generated.

**Table 1:** Top 3 stock performance by simulation period

Start date	End date	Stock	$\theta$	Annualized return(%)
2010	2012	LBV	1.007	18.459
2010	2012	CDM	1.011	13.217
2010	2012	MDP	1.052	10.106
2011	2013	LBV	1.007	14.731
2011	2013	JET	1.010	12.640
2011	2013	BMCI	1.012	12.204
2012	2014	MDP	1.026	16.947
2012	2014	LBV	1.014	16.725
2012	2014	ATH	1.012	15.098
2013	2015	COL	1.007	29.441
2013	2015	ATH	1.009	23.062
2013	2015	SNA	1.010	19.321

### Results

**Fig. 5:**
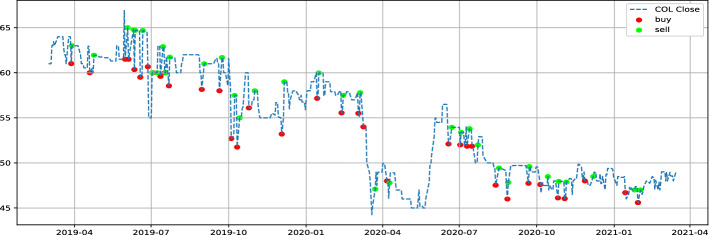
Buy and sell moment for COL stock

**Fig. 6:**
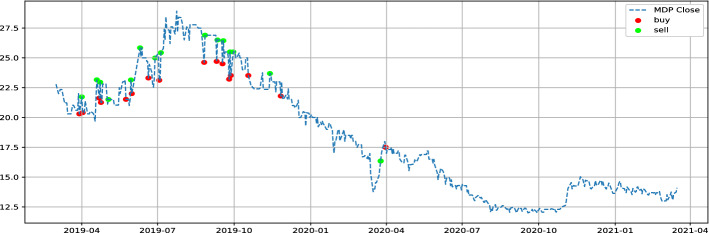
Buy and sell moment for MDP stock

**Fig. 7:**
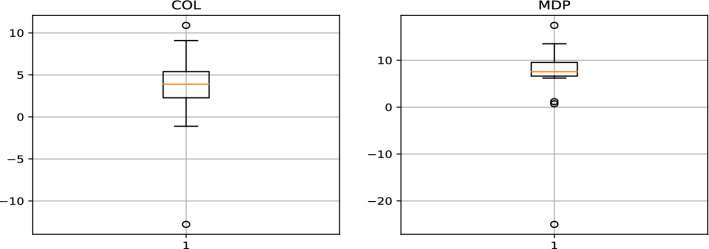
Performance per transaction (brokerage fees ignored)

COL (Colorado) and MDP (Med paper) are the stocks to keep in our watched list, with $\theta$ parameters of 1.01 and 1.03, respectively. In order to evaluate our proposed strategy performance, we will use data between March 2019 and March 2021. Table 2 show LSTM and GRU model prediction evaluation. Notice that no benchmark is used to evaluate prediction provided by models because it’s not the main scope of the paper, we are more focused on the proposed strategy performance. Table 3 illustrates the individual performance of our final stocks, while Figs. 5, 6 represent the buying and selling times for the same stocks respectively, and Fig. 7 highlight return generated per trade. In addition to individual stock results, the performance of our proposed strategy will be compared to the MASI index as well as the performance of the following sector indices: Distributors, Pharmaceuticals Industry, and Software & IT Services. Sector indices were chosen based on their results (annualized return $> 15\%$). Table 4 highlights this comparison. Notice that the decision to compare our results with indices results is justified by the lack of a publication dedicated to the Moroccan market. In addition, indices performance have always acted as a benchmark for evaluating performance of  financial product like UCITS.

**Table 2:** LSTM GRU performnces

Models	Stock	MAPE(%)	MSE	RMSE	Minimum price	Maximum price
LSTM	COL	1.96	2.25	1.5	44.26	66.9
LSTM	MDP	3.14	0.57	0.75	12	28.92
GRU	COL	2.12	2.81	1.67	44.26	66.9
GRU	MDP	4.30	0.84	0.91	12	28.92

**Table 3:** selected stock performance over period 03/2019 to 03/2021

Stock	$\theta$	Glboal return(%)	Annualized ret(%)	Winerate(%)	Number of closed position	Hloding time(days)
COL	1.01	57.80	26.87	91.43	35	4
MDP	1.03	64.02	29.46	93.33	15	14

**Table 4:** comparison of performance over the period from first March 2019 to first March 2021

Index or strategie	Monthly return (%)	Annualized return (%)
MASI	0.04	0.43
Distributors	1.19	15.24
Pharmaceutical industry	1.53	19.94
Software and computer services	3.34	48.30
Proposed strategy	2.02	27.13

## Discussion

**Fig. 8:**
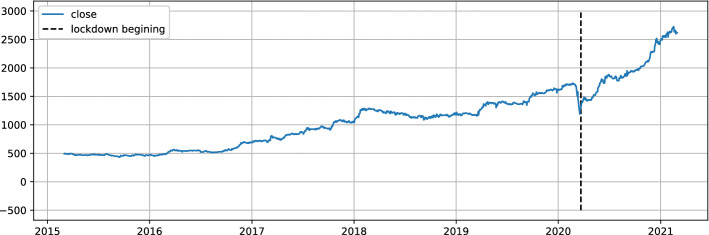
Historical data of software and computer services index

**Fig. 9:**
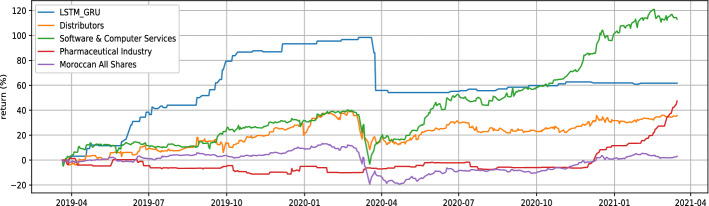
Performance comparaison over time

**Fig. 10:**
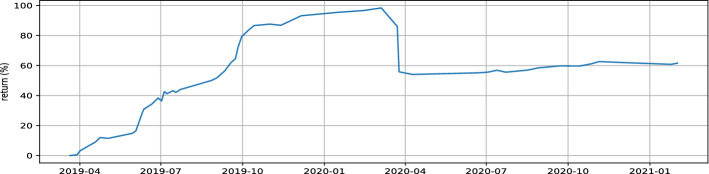
Proposed strategy return over time

Referring to Table 2, If we inspect the MAPE metric, the LSTM (short term prediction) model has 1.9% and 3.1%, respectively for the forecasting of the COL and MDP stock price. The GRU model (medium term prediction) has 2.1% and 4.3% for both stocks. Knowing that this is not a closing price forecasting, but rather a prediction of the moving average price for the next five days (resp 10 days for GRU). It is an excellent result because if we consider the closing price the difference will be even smaller. This finding is supported by the winerate Table 3 for our stocks (up to 91% both), which proves that the potential increase detected by the GRU or the decrease predicted by the LSTM are all relevant. For the same reason, the given MSEs and RMSEs are also worst-case indicators. Compared to the closing price their values will be even smaller. Referring to Table 3, the individual performance of the selected stock are perfectly aligned. Indeed, the amount invested at the start of the test period increase by 57% for COL and by more than 64% for MDP. A very strong observation for both stocks, the win rate ratio is very good, indeed 91% and 93% are quite high, indicating that our trade is winning most of the time. Fig. 7 is a visual form to observe the winerate ratio, we can see that most of the time the trade is winning in addition, 75% of the time the transaction return is greater than 2.5% for COL and greater than 5% for MDP. We also note the presence of some failure, a losing trade of 14% and 20% respectively for COL and MDP, the implementation of a security mechanism to limit losses can be an improvement. Despite this, the two stocks performance largely covers these losses and allows us to achieve a very satisfactory annualized return for our proposed strategy. From Table 4, the returns of our proposed strategy exceed the returns of all indices except Software & IT Services index. This index performance has risen dramatically because of the widespread use of remote applications during the COVD19 pandemic. Fig. 8 confirms this finding, the index is at its maximum never observed before during the pandemic phase. To better compare our strategy with the Software and Computer Services’ index and all other indices, Fig. 9 illustrates the return evolution for our proposed strategy as well as the other benchmark indices overall testing periods. We clearly observe the supremacy of our proposed strategy over the entire part prior COVID19 (22 March was the start of lockdown in Morocco). In fact, until April, our proposed method return was near 100%, while Software & IT Services’ index return does not exceed 40%. Distribution index return was also around 40%, while the rest of the indices were lower. The COVID19 crisis has halted the evolution of our proposed approach performance, indeed Fig. 10 shows a decrease in total return in March 2020. However, its return has not collapsed during this crisis. The overall result even during the crisis period remains very positive and it is a good point for our approach in terms of crisis management. Notice that pharmaceuticals, distribution, and information communication technology (ICT) are among the sectors not impacted by the COVID19 crisis, on the contrary, they are among the sectors that have gained from it. Despite this, only the ICT performance outperformed the result of our proposed strategy.

## Conclusion

This paper proposes a new trading strategy tailored for the Moroccan market, driven by two models (LSTM and GRU) forecasting. Customized decision rules for each stock complete the proposed trading strategy. Both deep learning models provide accurate short term and medium term forecasting. The proposed decision rules developed following varying simulations help detecting the potential price uptrend (resp downtrend) for the selected stocks (COL and MDP). The proposed approach allows selecting profitable stocks and the creation of portfolio that outperform all indices used as a benchmark except the Software & IT Services indices which witnessed an abnormal boom during COVID19 pandemic time. The proposed strategy is very promising and its performances are overall very satisfactory. Indeed, our proposed model provides an annualized return of 27.13% and a monthly return of 2.02%, whereas the best performance of a competing Indice (excluding Software & IT Services Indice) is 19.94% and 1.53% for annualized return and monthly return respectively. In our future work, we will continue to work on deep learning portfolio building techniques, but we will focus much more on the prediction of medium and long term horizon. We will also extend preprocessing part by incorporating natural language processing (NLP) techniques to capture the effect of social media, news, and rumors on the stock market price.

## Data Availability

Not applicable. For any collaboration, please contact the authors.
